# Sleep apnea: before and after heart transplant

**DOI:** 10.5935/1984-0063.20190120

**Published:** 2020

**Authors:** Elsa Matos, Teresa Calvo, Duarte Rocha, Ana Ferreira, Helena Moreira, Ana Mendes, Isabel Ferradosa, Ana Fernandes

**Affiliations:** Centro Hospitalar de Trás-os-Montes e Alto Douro, Pulmonology - Vila Real - Vila Real - Portugal

**Keywords:** Heart Transplantation, Sleep Apnea, Central, Sleep Apnea, Obstructive

## Abstract

Sleep disorder breathing is a highly prevalent public health problem and is common among patients with cardio and cerebrovascular diseases. Respiratory events are associated with numerous consequences, such as the hyperadrenergic state, known as a predictor of premature mortality in patients with heart failure. On the other hand, reduced stroke volume is associated with fluid retention in patients with heart failure, leading to changes in the upper airflow dynamics. Whether and how to treat sleep disorder breathing enables chronic cardiovascular consequences to be reversed is not fully established. Few cases are known where sleep disordered breathing diagnosis was made several years before heart transplantation. To better understand how does sleep apnea evolve and to ponder about what is the best treatment approach in this context, is the objective with this case presentation.

## INTRODUCTION

Sleep disordered breathing (SDB) is a high prevalent public health problem with numerous consequences^1^. Each respiratory event is associated with an increase of the negative intrathoracic pressure and hypoxic pulmonary vasoconstriction, and also aggravates cardiac hemodynamics. The effects of these repeated changes during the sleep period for several years are associated with the deterioration of cardiac function^2^^-^4.

Reduced stroke volume is associated with fluid retention in patients with heart failure (HF). Fluids are redistributed during sleep lying position, with a nocturnal fluid shift to the rostral sections of body. Peri-pharyngeal edema could happen, leading to changes of the upper airflow dynamics and favoring pharyngeal collapse^3^^-^5.

Concerning SDB treatment options in HF patients, the absence of treatment might promote the progression of cardiac disease. Treatment with continuous positive airway pressure (CPAP) improved cardiac function in HF patients with central sleep apnea (CSA) by stabilizing ventilation. However, CPAP use could have been limited in some cases due to reduced efficacy and intolerance. For this reason, large trials used adaptative servo-ventilation (ASV) instead of CPAP, since this ventilatory mode effectively suppresses CSA and is well tolerated^4^^,^^6^.

Treatment options for SDB are evolving. In general, however, CSA is more difficult to treat than obstructive sleep apnea (OSA). Several positive airway pressure (PAP) devices, including CPAP, bilevel and ASV have been tested. ASV equipment has been shown to be useful, however, the SERVE-HF trial cast doubts on this notion after the 2015 SERVE-HF study^7^.

Early case reports suggest that normalization of heart function after transplantation leads to complete abolition of CSA or the development of OSA^8^. Nevertheless, CSA persisting within a few days after heart transplantation (HT) has also been reported^9^. In a prospective controlled trial of 22 patients with final stages of HF, sleep apnea (SA) severity was reduced after surgery in individuals presenting CSA- Cheyne stokes respiration (CSR) before HT. Nonetheless, SA did not disappear after HT and obstructive events were the predominant type in most patients^4^^,^^9^.

The expectation is that restoring cardiac function with HT might change the clinical history of CSA-CSR. Few cases are known where SA diagnosis was made several years before HT. The objective with this case presentation is to better understand how SDB evolves and to ponder with the best treatment approach in this context.

## CLINICAL CASE

Male patient, currently 67 years old, non-smoker and with normal body mass index (BMI). Followed through cardiology consultation at another institution since 1994 due to congenital atrio-ventricular block and aortic disease. The patient has been submitted to definitive pacemaker (PM) (DDD) implantation in April 2002. At that time, had mild to moderate degree of aortic insufficiency with normal ventricular dimensions and preserved left ventricular (LV) systolic function (SF). 

**Figure 1:**
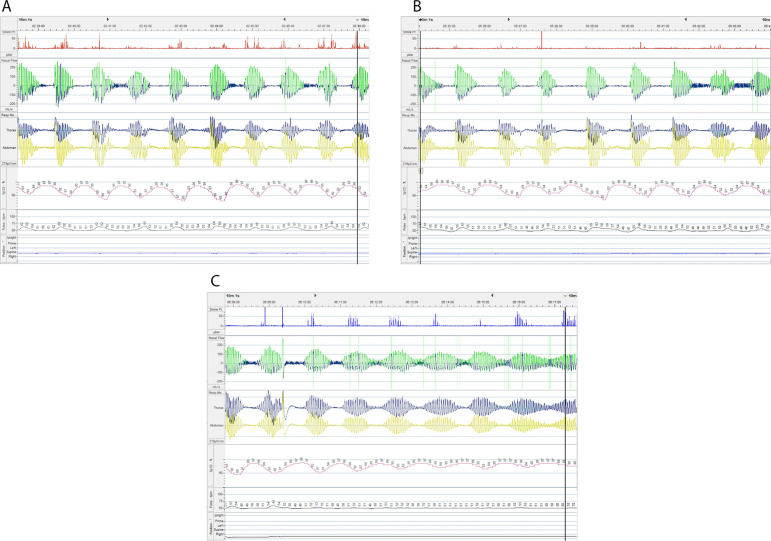
A, B, C. Diagnostic test - Home respiratory polygraphy performed in July 2007: respiratory pattern with recurrent central apneas and Cheyne Stokes pattern.

A sleep polygraphy was performed in July 2007 for reported snoring, frequent dreams, waking up with dry mouth and respiratory pauses (Epworth Sleepiness Scale: 5). The exam revealed an apnea/hypopnea index (AHI) of 28.1/h and central apnea index (CAI) of 8.3/h (Figure 1). Due to the presence of HF and CA with suggestive CSR, the ASV therapy was instituted with oronasal mask.

Between 2007 and 2008, the patient was always well adapted to ASV. In August 2008 there was a clear LV function deterioration due to the aortic disease progression; the echocardiogram presented severely dilated LV with severely depressed global SF (LV ejection fraction (LVEF) estimated at 25%) and moderate aortic insufficiency with eccentric jet directed at the anterior leaflet of the mitral valve.

Since it was an end-stage of HF, the individual was observed at a thoracic surgery consultation in September 2009. Medical treatment was maintained because the patient was considered not to meet conditions for classic aortic valve replacement surgery.

Re-evaluation in September 2010 considered the individual a candidate for HT if the functional class aggravates. A reassessment by polysomnography (PSG) was made in February 2011 showing a respiratory disturbance index (RDI) of 14.2/h.

In April 2011 the cardiac catheterization revealed absence of coronary disease, but severe aortic insufficiency with severely depressed LVEF; pulmonary vascular resistance (PVR) of 5.67 U/Wood, with pulmonary arterial systolic pressure (PASP) of 70 mmHg and pulmonary capillary wedge pressure (PCWP) of 37 mmHg.

In July 2011, given that it was time to replace the PM generator, it was decided to perform the upgrade to cardiac resynchronization therapy (CRT), to attempt to compensate for the cardiac insufficiency. The procedure was unsuccessful after a small dissection of the coronary sinus when trying to position the left ventricular probe. At that time, the echocardiogram revealed more marked dilated cardiomyopathy (LVEF 23%); severe aortic insufficiency due to prolapse of the right coronary cusp, conditioning an eccentric jet directed to the anterior leaflet of the mitral valve. ERROR - 0.52 cm^2^; holodiastolic reverse flow in the descending aorta and slightly dilated right ventricle (RV) with compromised systolic function.

The patient was electively hospitalized in September 2011 for attempted CRT implantation, which occurred without complications. Repeated evaluation of the pulmonary resistances showing a clear improvement in the parameters (PVR - 1.74 U/Wood; PASP - 34 mmHg; PCWP - 14 mmHg). Therefore, it was decided to initiate a pre-transplant cardiac study: performed abdominal ultrasound and renal-vesicle-prostatic ultrasonography demonstrating prostatic hypertrophy (already known) and complex cyst in the right kidney (classified as a lesion Bosniak IIF). Lung function tests were normal; serology for infections and vaccination was done; got indication to complete dental treatment; was also evaluated for infectious diseases and psychiatry consultations and histocompatibility study was performed.

In November 2011, an echocardiogram revealed severe dilation of the left heart chambers (LA 90 cm^3^, LVtd 80 mm); severe aortic insufficiency due to prolapse of the right coronary cusp conditioning an eccentric jet directed to the anterior leaflet of the mitral valve; non-dilated inferior vena cava presenting variability of about 50%; LV global hypokinesia with severely compromised systolic venous function, EF of 18%; compromised SF of the RV; electrocatheters were in the right cavities.

Regarding the sleep consultations, all the 5 evaluations were made between 2011 and 2014, with approximately 100% of ASV utilization, and residual AHI<5/h, with average usage higher than 8h/night (in 2013 for referring nasal obstruction was sent to otolaryngologist consultation).

The patient underwent HT in September 2015 (description of the intervention: orthotopic HT by binaural technique, removal of the PM and its probes, postoperative post-discharge at day 36). After HT was evaluated in the sleep consultation in March 2016, the individual continued with ASV by stating not being able to sleep without it. Adherence of 97% and residual AHI of 0.6/h. Approximately one year later, the patient maintains excellent adherence to the treatment.

**Figure 2:**
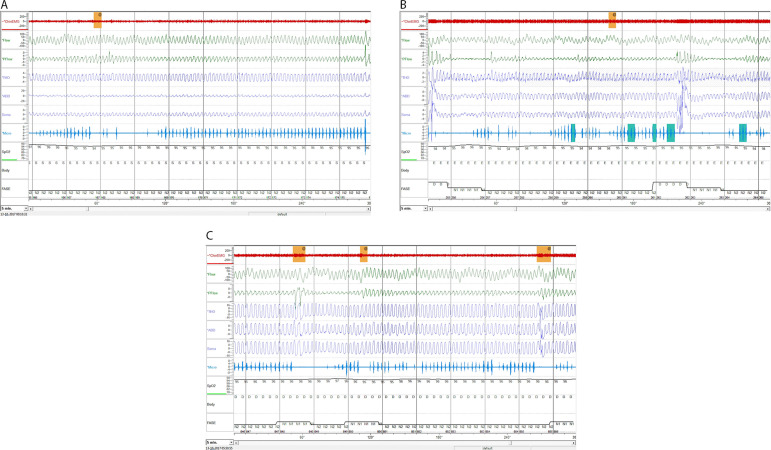
A, B, C. Revaluation sleep test (after heart transplantation). Overnight polysomnography performed in October 2017: respiratory pattern with occasional respiratory effort related arousals.

During 2017, a revaluation sleep study revealed an RDI of 16.9/h with CAI of 0/h (Figure 2). The echocardiogram showed preserved biventricular function (EF of 74%); good RV SF and tissue Doppler imaging (TDI) S wave - 0.12 m/s. Given de absence of CA, in March 2018, despite excellent adhesion to ASV, the sleep specialist decided to suspend the therapy. Automatically-adjusting positive airway pressure (APAP) was prescribed (pressures: 4-16 cmH_2_O; nasal mask).

In April 2018, card reading showed AHI<5/h, 73% usage > 4h and P95% of 11.6 cmH_2_O. A change to CPAP mode was programmed (based on APAP P95).

The last echocardiogram information, from July 2018, showed: good left LV global SF (estimated EF: 61%); abnormal movement of the interventricular septum; good RV SF and TDI S wave - 0.11 m/s. Since HT always has dysthymic dysfunction, the cardiologist was of the opinion that it should continue with CPAP because it does not have CA. The patient continued well adapted to CPAP, referring to having good sleep quality until March 2019.

## CONCLUSION

International guidelines recommend the diagnosis and treatment of SDB in HF patients due to their bidirectional relationship. 

After HT, treatment with ASV mode -although no formal indication exists- seems to have been an acceptable ventilatory mode since there was no impact on cardiac function, keeping respiratory events and symptomatology controlled. We insisted on switching to CPAP mode since it only had obstructive events. 

Therapeutic implications of treating SDB in HF patients and treating HF in patients with SDB, remains to be fully elucidated. SDB treatment with PAP in HF patients should be started cautiously checking for possible hemodynamic changes and involving a multidisciplinary team.
